# E-cigarette flavored pods induce inflammation, epithelial barrier dysfunction, and DNA damage in lung epithelial cells and monocytes

**DOI:** 10.1038/s41598-019-51643-6

**Published:** 2019-12-13

**Authors:** Thivanka Muthumalage, Thomas Lamb, Michelle R. Friedman, Irfan Rahman

**Affiliations:** 1 Department of Environmental Medicine, University of Rochester Medical Center, School of Medicine & Dentistry, Rochester, NY USA; 2 Department of Chemistry & Biochemistry, College of Brockport, State University of New York, NY New York, USA

**Keywords:** Acute inflammation, Chemokines

## Abstract

E-cigarette flavored pods are increasing in use among young adults. Although marketed as a safer alternative to conventional cigarettes, the health effects of e-cigarette flavored pods are unknown. We hypothesized that e-cigarette flavored pods would cause oxidative stress, barrier dysfunction, and an inflammatory response in monocytes and lung epithelial cells. JUUL pod flavors (Fruit Medley, Virginia Tobacco, Cool Mint, Crème Brulee, Cool Cucumber, Mango, and Classic Menthol) and similar pod flavors (Just Mango-Strawberry Coconut and Caffé Latte) were tested. These pod flavors generated significant amounts of acellular ROS and induced significant mitochondrial superoxide production in bronchial epithelial cells (16-HBE). Lung epithelial cells (16-HBE, BEAS-2B) and monocytes (U937) exposed to various pod aerosols resulted in increased inflammatory mediators, such as IL-8 or PGE_2_. JUUL pod flavors, Crème Brulee and Cool Cucumber, caused epithelial barrier dysfunction in 16-HBE cells. Moreover, tested flavors also showed DNA damage upon exposure in monocytes. We determined the chemical constituents present in various flavors. Our data suggest that these constituents in flavored pods induce oxidative stress, inflammation, epithelial barrier dysfunction, and DNA damage in lung cells. These data provide insights into the regulation of e-cigarette flavored pods, as well as constituents in these flavors.

## Introduction

Electronic cigarettes or e-cigarettes (ENDS, electronic nicotine delivery system) are battery-powered devices that can generate an aerosolized vapor from a liquid typically consisting of nicotine, propylene glycol (PG), vegetable glycerin (VG), and flavoring chemicals. Currently, there is a great diversity in flavorings, with over 8000 different flavors available in the market^1^. These flavors are generally regarded as safe for ingestion, but inhalation toxicity has yet to be determined^2^. Flavoring chemicals from e-cigarettes have previously been shown to cause inflammatory responses in lung epithelial cells and monocytes mediated by oxidative stress caused by reactive oxygen species (ROS) generation^3,4^.

E-cigarette use is currently on the rise in the western population, such as the United States, with an increase in the prevalence of use among high-schoolers and middle-schoolers. The use of e-cigarettes in high-schoolers and middle-schoolers was increased by 78% and 48% from 2017 to 2018, respectively^5^. One e-cigarette of particular interest is JUUL, since as of July 2018, JUUL devices account for 70.5% of US convenience store vapor product sales^6^. Using e-cigarettes is considered a harm-reduction approach for traditional tobacco smoking. Nevertheless, moderate nicotine dependence has been reported in a community-based study with correlating levels of salivary cotinine^7^. According to a survey-based study, 62.9% of e-cig users expressed a greater satisfaction from vaping flavored e-cigs, sweet, fruity, and minty flavors, compared to tobacco or unflavored e-cigs^8^.

These pod mod devices with USB charging capability and alluring flavors is a contributing factor to initiate vaping, especially among the youth^8,9^. JUUL pods are comprised of a heat resistant plastic enclosing the atomizer with a stainless steel vapor path, silica wick, and nichrome coil heater **(**Supplementary Fig. S1A**)**. JUUL uses pre-filled pods consisting of PG, VG, nicotine, benzoic acid, and flavoring chemicals^6^. Benzoic acid and nicotine in these pods are likely to form nicotine benzoate during aerosolization^10^. Recent findings determined that average nicotine concentration was 60.9 mg/mL, levels that match the nicotine concentration of a pack of cigarettes^11^. The major flavoring chemicals found in JUUL pods are menthol, vanillin, and ethyl maltol^11^. There have been some cytotoxic effects seen from these flavoring chemicals, with menthol and ethyl maltol to cause oxidative stress, an inflammatory response, and barrier dysfunction, and vanillin to be a respiratory irritant which induces an inflammatory response^2^. Exposure of the airway epithelium to environmental toxicants such as JUUL pod chemical constituents, metals, and ROS can lead to disruption of the barrier junctions allowing hazardous agents to enter into subepithelial tissues inducing lung damage, repair, immunomodulation, and disease progression. The etiology of respiratory diseases such as asthma, bronchitis, bronchiolitis obliterans is the exposure to environmental insults, such as environmental tobacco smoke and airborne particulates. Hence, it is conceivable that chronic exposure to JUUL pods and similar pod aerosols potentially have similar risks, especially in developing lung.

Although JUUL pod is typically marketed as an alternative to conventional cigarette smoking with reduced harmful effects, the effects of these devices on the individuals using them are still relatively unknown. We hypothesized that flavorings used in e-liquids, JUUL pods, and other pod flavors would result in oxidative stress and inflammatory responses in lung epithelial cells and monocytes and cause airway epithelial barrier dysfunction.

## Methods

### Scientific rigor and reproducibility

We used a rigorous and unbiased approach throughout the experimental plans and data analyses to ensure that our data are reproducible along with full and detailed reporting of both methods and analyzed data. All the key biological and chemical resources that are used in this study were validated and authenticated and are of scientific standard from commercial sources. Our results adhere to NIH standards of reproducibility and scientific rigor.

### Ethical approval: Institutional biosafety approvals

All experiments performed in this study were approved and in accordance with the University of Rochester Institutional Biosafety Committee. Cell lines (16-HBE, BEAS2B, U937) were procured from ATCC, USA. No ethical approval is required for these cell lines.

### Procurement of JUUL pods and similar pod flavors

JUUL pod flavors, “Fruit Medley”, “Virginia Tobacco”, “Cool Mint”, Crème Brulee”, “Cool Cucumber”, “Mango”, and “Classic Menthol” with 5% nicotine were purchased from the JUUL online store as well as the local retail store. Other pod flavors, “Just Mango (Strawberry Coconut)” (nicotine concentration unlisted) by LCF labs and “Caffé Latte” by Eonsmoke labs with 6% nicotine, were purchased from local vape shops in Rochester, NY.

### Qualitative analysis of the composition of JUUL pod constituents

JUUL pod flavors (Fruit Medley, Classic Menthol, Cool Mint, Crème Brulee, Cool Cucumber, and Virginia Tobacco) were diluted 100X into spectral grade methanol and injected into the gas chromatograph (GC) with a mass spectrometer (MS) detector (Agilent 7890 A gas chromatograph with 5975 MSD detector). The system used helium as the carrier gas, flowing 1.2 mL/min through an Agilent Technologies column (HP-5MS, 30 m × 0.250 mm, 0.25 μm, 19091S-433). The oven program initiated at 60 °C for 4 minutes, ramped to 150 °C over 2 minutes at 70 °C/min, spiked to 250 °C and then ramped again to 285 °C over 5 minutes at 25 °C/min. The total run time was almost 18 minutes, and the injection volume was 1 µL from a 10 µL syringe. The samples were analyzed by electron impact ionization in positive ion mode with a mass range of 50–550 m/z, with the source temperature at 230 °C and the quadrupole at 150 °C. Data analysis was performed using Agilent ChemStation software, with ion scans searched against the NIST database for identification. Retention times with the percentage probability of the constituents found are listed in Supplementary Table S1.

### Assessment of acellular ROS

For the acellular ROS assessment, 2′7′dichlorofluoroscien diacetate (DCFH-DA) dye (Calbiochem, CA, catalog #287810) was prepared by mixing 5 mM DCFH-DA with 0.01N NaOH and followed by the addition of 25 mM phosphate buffer and HRP-streptavidin thirty minutes later. JUUL pod flavor vapors from the seven pod flavors were bubbled in the DCFH dye by a SCIREQ air pump following a puff profile of three puffs per minute lasting for three seconds with roughly 17s intervals at a flow rate of 1.6 L/min. For each pod, there were five, ten, or fifteen puffs bubbled through the DCFH dye. Using a standard curve generated from various H_2_O_2_ concentrations, produced ROS concentrations were obtained in μM H_2_O_2_ equivalents for each flavor.

### Cell culture and flavor exposure/treatment

#### Human bronchial epithelial cells, 16-HBE cell line

16-HBE at 20,000 cells per well were cultured in 24-well transwell inserts (Corning, #3470) in complete media (DMEM media with 10% FBS, 15 mM HEPES, 1% Pen/strep, and 0.2% amphotericin B). At 80% confluency, the cells were serum-deprived at 1% FBS for 12-hours followed by three-sessions of aerosol exposure to Cool Cucumber, Classic Menthol, Just Mango (Strawberry Coconut), and Caffé Latte flavors (refer to the *in vitro* exposure section for more details on exposure methods). Treatment with H_2_O_2_ (600 μM) was used as a comparison positive control. Immediately after the aerosol exposure, the cells were used for the barrier dysfunction assessment and MitoStress assay. The conditioned media was stored at −80 °C for cytokine assessment with ELISA and Luminex.

#### Human bronchial epithelial cells, BEAS-2B cell line

BEAS-2B cells were cultured in 6-well plates with approximately 325,000 cells per well in complete media in DMEM: F12 complete media with 5% FBS, 1% pen/strep, and 15 mM HEPES. At 80% confluency, the cells were serum-deprived at 1% FBS for 12-hours followed by three sessions of aerosol exposure to Cool Cucumber, Classic Menthol, Just Mango (Strawberry Coconut), and Caffé Latte flavors (refer to the *in vitro* exposure section for more details on exposure methods). Tumor necrosis factor alpha (TNF-α) (10 ng/mL) treatment was used as a comparison positive control. Twenty-four hours post aerosol exposure, the conditioned media were collected and stored at −80 °C for cytokine assessment with ELISA and Luminex multiplex assay.

#### Monocytes, U937 cell line

U937 cells (approx. 300,000) were cultured in 12-well plates in complete RPMI 1640 media (ATCC, 30-2001) with 10% FBS complete with 1% pen/strep. Approximately 12-hours before treatments cells were serum-deprived at 1% FBS. Subsequently, designated wells were directly treated with Cool Cucumber, Classic Menthol, Just Mango (Strawberry Coconut), Caffé Latte flavor liquids at 0.5% and H_2_O_2_ (100 µM) as a positive control. Twenty-four hours post-exposure the conditioned media was used for cytokine analysis by ELISA and Luminex, and the cells were used for DNA damage assessment by Comet assay.

#### *In-vitro* aerosol exposure system

JUUL pod device was connected to one of the pumps of the Scireq Inexpose e-cig exposure system (Scireq, Monreal). The mouthpiece of the JUUL device was then connected to an Enzyscreen chamber (Enzyscreen, Netherlands). For each exposure session, a cell culture plate was placed inside the Enzyscreen chamber and the vapors were released into the chamber. (66 puffs during 22 minutes with a three second puff duration at 1.6 L/min flow rate and an interpuff interval of approximately 17s.). The aerosols were allowed to equilibrate for eight minutes post-exposure resulting in a total of 30 minutes per session before returning the cell culture plate to the incubator. Next, aerosol exposure sessions were performed at 12-hour intervals **(**Supplementary Fig. S1B**)**.

#### Assessment of mitochondrial superoxide generation

Immediately after the last exposure with the selected flavor aerosol, the cells were trypsinized and stained with MitoSOX Red and Annexin V according to MitoStress kit protocol (Millipore, FCCH100109). For the flavors, Cool Cucumber, Classic Menthol, Just Mango (Strawberry Coconut), and Caffé Latte, six transwells were pooled together per flavor, and two pooled samples were run for each tested flavor. Sample acquisition and analysis were performed by the Guava Millipore Easycyte-8 instrument and its software, respectively.

#### IL-8 cytokine quantification by ELISA

Conditioned media from 16-HBE and U937 cells were collected after the specified incubation period post-treatment/exposure, and the IL-8 levels were quantified using ELISA kit (Invitrogen, CHC1303) according to the manufacturer’s instructions.

#### Prostaglandin E_2_ quantification by ELISA

Conditioned media from U937, BEAS-2B, and 16-HBE cells were collected after the specified incubation period post-treatment/exposure. A competitive ELISA assay was performed to quantify PGE_2_ levels according to the manufacturer’s protocol (Cayman, 514010).

#### Quantification of inflammatory mediators by Luminex

Conditioned media collected from BEAS-2B and U937 cells were assayed for inflammatory cytokines and growth factors by Bioplex Pro human cytokine 27-plex (BioRad, M500KCAF0Y). After the specified post-exposure/treatment incubation period, collected conditioned media was assayed for cytokines with Bioplex 3D system. Only the detectable mediators were plotted and compared with the untreated control group.

#### Epithelial barrier function assessment

After 16-HBE cell exposure to Crème Brulee, Classic Menthol, Just Mango (Strawberry Coconut), and Caffé Latte flavors, the barrier function was evaluated by EVOM2 (WPI instruments, FL). The transepithelial voltage and the resistance were measured three times in each transwell at different positions, and the average numbers were reported per transwell.

#### DNA damage assessment by JUUL pods and other pod flavors

Twenty four hours after monocytes (U937) being treated with JUUL pods and similar pod flavors, the cells were prepared for DNA damage assessment by Comet assay according to the manufacturer’s protocol (Trevigen, MD). Briefly, the alkaline procedure was followed and approximately 5000 cells per sample were used and stained with SYBR gold 1:10,000. EVOS imaging system was used to capture the fluorescence images. The images were inverted for the Opencomet analysis. The olive tail moment was obtained using Opencomet software for the representative images, and the percent change was calculated in comparison to the control group.

### Statistical analysis

Statistical analysis of data was done using two-way ANOVA with a Tukey’s *post-hoc* test for all analyses of multiple groups with multiple variables and one-way ANOVA with Dunnett’s *post-hoc* test was used to analyze all comparison of multiple groups with a single variable using GraphPad Prism 8.0. Data are represented as mean ± SEM. Cells and conditioned media samples were pooled in some assays. Statistical significance was reported as *p < 0.05, **p < 0.01, and ***p < 0.001.

### Disclaimer

The authors have nothing to claim or disclaim about any products used here to test their toxicological and biological effects. The authors have no personal interests or gains from the outcome of this study. The products tested are available commercially to consumers/users.

## Results

### JUUL pod chemical constituents

Assessment of JUUL pod constituents by GC-MS showed the presence of numerous flavoring chemicals and flavor-enhancing chemicals. Nicotine was present in all tested flavors, except Cool Mint **(**Supplementary Table S1).

### Acellular (cell-free) ROS generated by JUUL pod flavors

**Figure 1:**
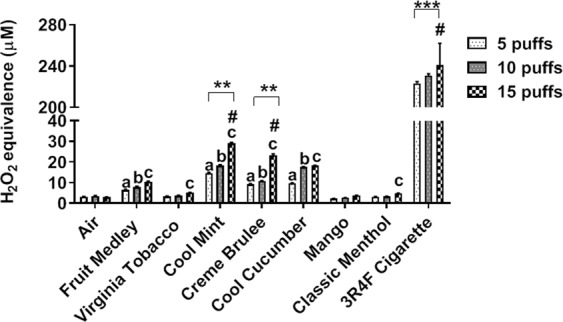
Cell-free ROS generated by JUUL pod flavors. JUUL pods containing Fruit Medley, Virginia Tobacco, Cool Mint, Cool Cucumber, Mango, and Classic Menthol flavors and 3R4F research cigarettes were bubbled through DCFH fluorescent dye. Filtered air was bubbled as a negative control comparison group. Five, ten, and fifteen puffs were generated of these flavors each puff with three-second puff durations. ROS levels are expressed in H_2_O_2_ µM equivalents. ^a^p < 0.001 compared to 5 puff air control, ^b^p < 0.001 compared to 10 puff air control, and ^c^p < 0.001 compared to 15 puff air control, 3R4F research cigarettes were not analyzed; **p < 0.01, and ***p < 0.001 vs. 5 puffs ^#^p < 0.001 vs. 10 puffs. Two wells per group with repeated measurements in pooled samples.

To determine the ROS generation by JUUL pods, the vapors were drawn through DCFH-DA dye and the fluorescence was measured in H_2_O_2_ equivalents. JUUL pod flavors, Cool Mint, Crème Brulee, Cool Cucumber, and Fruit Medley, generated significantly higher ROS levels at each puff count compared to the respective air control group. The vapors generated from the JUUL pods followed a dose-dependent increase in ROS with an increasing number of puffs, with both Cool Mint and Crème Brulee having a significant increase between 10 and 15 puffs and 5 and 15 puffs **(**Fig. 1**)**.

### Increased mitochondrial superoxide generation due to JUUL pods and other pod flavors

**Figure 2:**
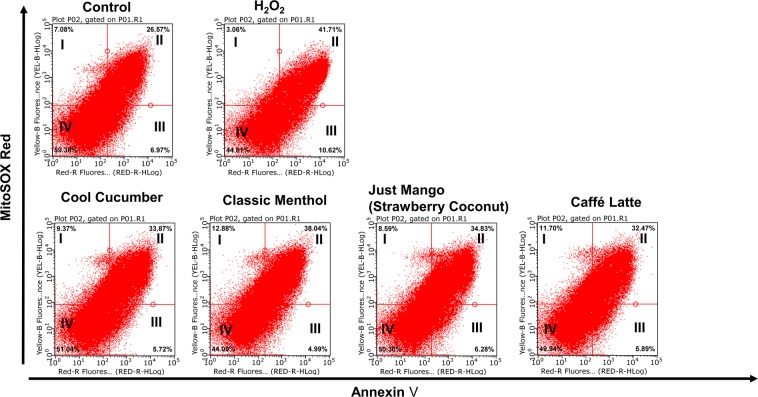
JUUL pods and other pod flavors induce the production of mitochondrial superoxide in lung epithelial cells. 16-HBE cells with 20,000 cells per transwell were grown to 80% confluency in complete media. After serum deprivation (1% FBS) the wells were exposed to three-sessions (each session 66 puffs) of JUUL pods and other pod aerosols with equal intervals between each session. Aerosolized flavors included JUUL pods (Classic Menthol and Cool Cucumber) and other pods (Just Mango -Strawberry Coconut and Caffé Latte). Air group and H_2_O_2_ (600 μM) were used as assay controls. After the last exposure, cells were collected and pooled for each treatment group (n = 3–6 pooled transwells per group) and stained with MitoSOX red and annexin V, and flow-cytometry analysis was performed. Representative scatter plots per treatment group were shown.

To determine cellular oxidative stress by JUUL pods and other pod aerosols were assessed by staining MitoStress assay. Each sample was analyzed by dividing the scatter plots into 4-quadrants, I, II, III, and IV, with each quadrant representing a cell population **(**Fig. 2**)**. Quadrant I depicts the cell population MitoSOX high and Annexin V low population, quadrant II depicts MitoSOX high and Annexin V high cell population, quadrant III depicts MitoSOX low and Annexin V high cell population, and quadrant IV with MitoSOX low and Annexin V low population. With the H_2_O_2_ positive control treatment, there was a definitive shift in the population towards quadrant II, demonstrating increased mitochondrial superoxide production as well as increased cell death compared to the untreated control group. Similarly, Cool Cucumber, Classic Menthol, Just Mango (Strawberry Coconut), and Caffé Latte pod flavors showed on average a 28% shift in the cell population toward quadrant II. These data show that exposure to tested flavors increased mitochondrial superoxide production with Classic Menthol inducing the greatest mitochondrial ROS production with 12.88% and 38.04% in quadrants I and II, respectively. Cell death due to JUUL pod aerosols exposure (quadrant V) is under 8%. These data demonstrate exposure to JUUL pod aerosols induces marked mitochondrial ROS production and may result in negligible cell death.

### Increased IL-8 inflammatory cytokine response in 16-HBE cells

**Figure 3:**
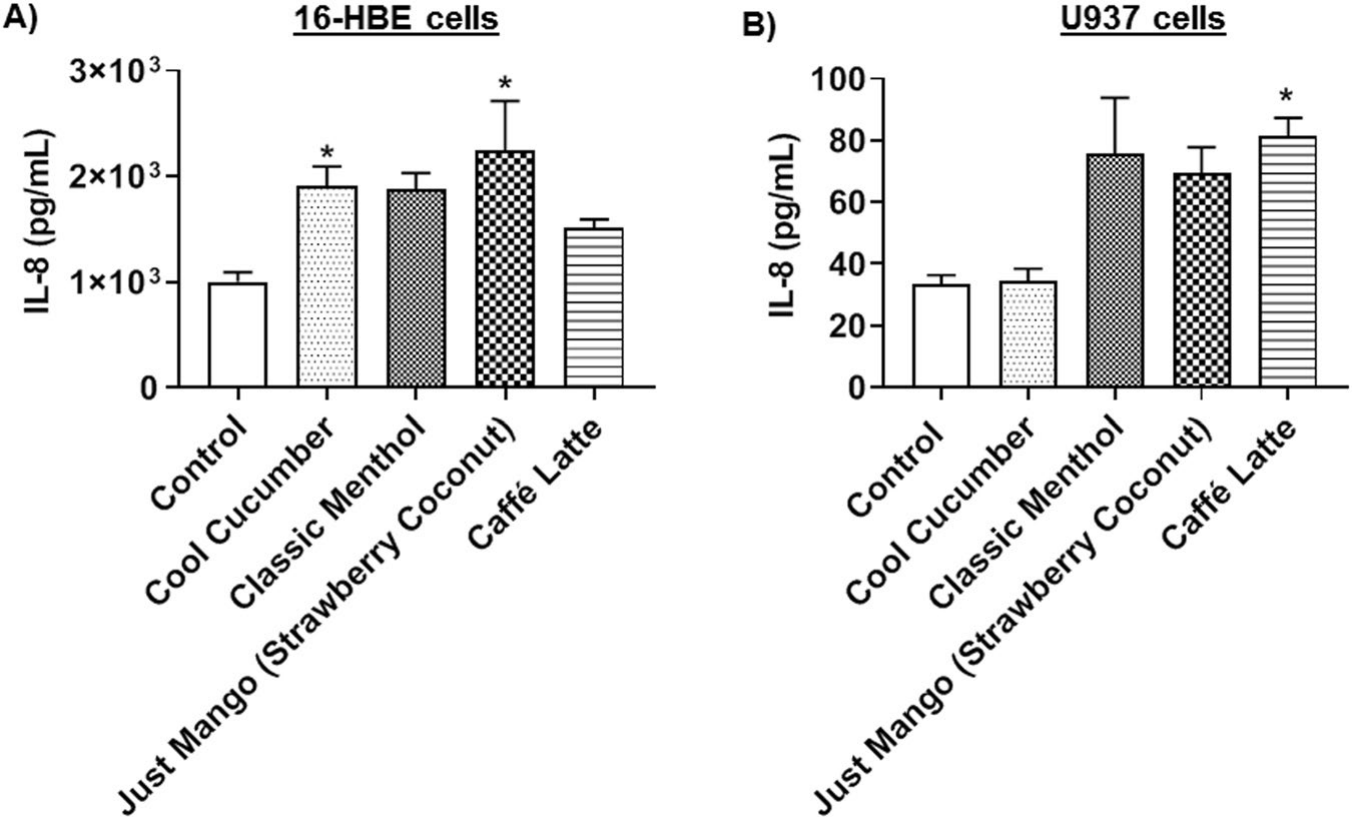
JUUL pods and other pod flavors induce the production of IL-8 in lung epithelial cells and monocytes. (**A**) 20,000 16-HBE cells grown in 24-well transwell plates exposed to three-sessions (each session 66 puffs) of JUUL pods and other pod aerosols with equal intervals between sessions. Aerosolized flavors were JUUL pods (Cool Cucumber and Classic Menthol) and other pod flavors (Just Mango -Strawberry Coconut and Caffé Latte). After the last exposure, IL-8 was measured in the conditioned media. *p < 0.05 vs. Control. N = 3 wells per group. (**B**) Approximately 300,000 U937 cells were cultured in a 12-well plate in complete medium. Cells were serum-deprived (1% FBS) for 24 hours and treated with 0.5% JUUL pods and other pod flavors. Treated flavors included JUUL pods (Cool Cucumber and Classic Menthol) and other pod flavors (Just Mango-Strawberry Coconut and Caffé Latte). Twenty-four hours later, IL-8 levels in the conditioned media were determined. *p < 0.05 vs. Control.

To determine the potential to elicit an inflammatory response, 16-HBE cells were exposed to various JUUL pods and other pod flavors, and the IL-8 production was measured in the conditioned media. JUUL pod flavor, Cool Cucumber significantly increased the IL-8 levels compared to the unexposed group. Similarly, Just Mango (Strawberry Coconut), also secreted significantly high IL-8 levels compared to the untreated control group **(**Fig. 3A**)**.

### Increased IL-8 inflammatory cytokine response in monocytes

Monocytes directly treated with Classic Menthol flavor JUUL pod liquid showed an increase in IL-8 levels, albeit not significant. Treatment with the pod liquid, Caffé Latte, showed a significant increase in IL-8 compared to the untreated control group **(**Fig. 3B**)**. Individual treatments with Classic Menthol and Caffé Latte showed significant cytotoxicity in monocytes, with a 34% and a 67% decrease in cell viability compared to the untreated, respectively, whereas all other treatments showed insignificant cytotoxicity (data not shown).

### Increased prostaglandin E_2α_ (PGE_2_) response in monocytes cells by JUUL pods and other pod flavors

**Figure 4:**
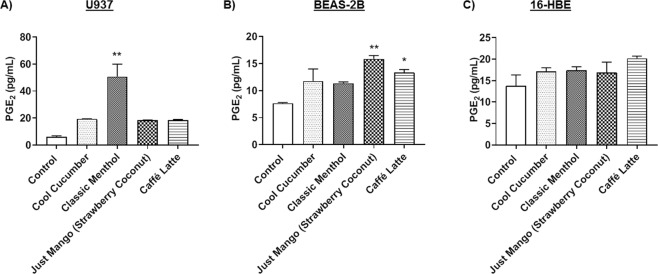
JUUL pods and other pod flavors induce the production of PGE_2_ in lung epithelial cells and monocytes. (**A**) U937 with 300,000 cells per well were treated with 0.5% JUUL pods and other pod flavors in serum-deprived medium. Flavors included JUULpods (Cool Cucumber and Classic Menthol) and other pod flavors (Just Mango-Strawberry Coconut and Caffé Latte). Prostaglandin E_2_ was measured in conditioned media. **p < 0.01 vs. control. (N = 2 treated wells per group with repeated experiments). (**B**) Approximately 325,000 BEAS-2B cells were cultured in 6-well plates and exposed to JUUL pods and other pod flavors in serum-deprived medium (1% FBS). Three sessions of 66-puffs were generated by Scireq Inexpose system. Aerosolized flavors included JUUL pods (Cool Cucumber and Classic Menthol) and other pods (Just Mango - Strawberry Coconut and Caffé Latte). Prostaglandin E_2_ was measured in conditioned media. *p < 0.05, **p < 0.01 vs. control. N = 2–6 treated wells per group. (**C**) 20,000 16-HBE cells cultured in 24-well transwell inserts and exposed to JUUL pods and other pod flavors in serum-deprived medium (1%). Three sessions of 66-puffs were generated by Scireq Inexpose system. Flavors included JUUL pods (Cool Cucumber and Classic Menthol) and other pods (Just Mango-Strawberry Coconut and Caffé Latte). Prostaglandin E_2_ was measured in conditioned media. N = 3–12 treated wells per group with repeated experiments.

Monocytes (U937) were directly treated with Cool Cucumber, Classic Menthol, Just Mango (Strawberry Coconut), and Caffé Latte. Among the tested pod flavors, only Classic Menthol treatment significantly increased the PGE_2_ levels compared to the untreated control **(**Fig. 4A**)**.

### Increased Prostaglandin E_2α_ (PGE_2_) response in epithelial cells by JUUL pod and other- pod flavors

BEAS-2B epithelial cells exposed to JUUL pod flavors, Cool Cucumber and Classic Menthol, did not show a significant increase in PGE_2_ levels in conditioned media even though the levels were increased with the exposure. Just Mango (Strawberry Coconut) and Caffé Latte showed significantly elevated levels of PGE_2_ compared to the unexposed control group **(**Fig. 4B**)**. 16-HBE cells, on the other hand, showed no significant changes in PGE_2_ levels compared to the control group **(**Fig. 4C**)**.

### Differential inflammatory response due to JUUL pods and other pod aerosols in epithelial cells

**Figure 5:**
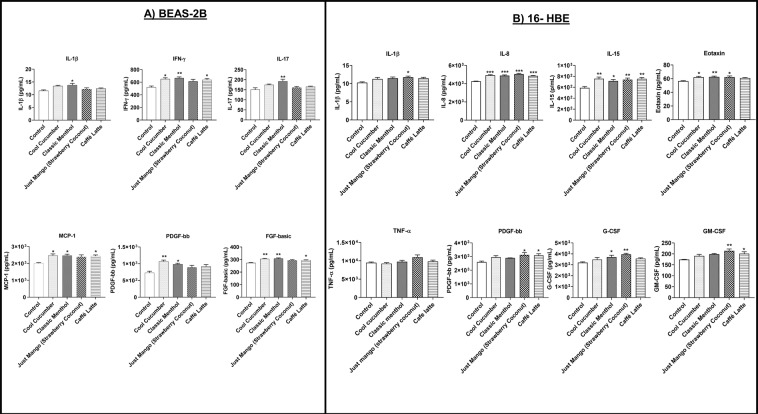
JUUL pods and other pod flavors produce differential effects on inflammatory mediators and growth factors in lung epithelial cells. (**A**) BEAS-2B cells were grown and exposed to three sessions (66 puffs per session) of JUUL pods and other pod aerosol with equal intervals between sessions. Aerosolized flavors were JUUL pods (Cool Cucumber and Classic Menthol) and other pods (Just Mango-Strawberry Coconut and Caffé Latte). The air control group was used as the negative control. After the last exposure condition media was measured using Luminex for inflammatory cytokines and growth factors. *p < 0.05, **p < 0.01 vs. control, N = 2–6 wells per group. (**B**) 16-HBE cells were grown and exposed to three sessions (66 puffs per session) of JUUL pods and other pod aerosols with equal intervals between sessions. Aerosolized flavors were JUUL pods (Cool Cucumber and Classic Menthol) and other pods (Just Mango-Strawberry Coconut and Caffé Latte). The air group was used as the negative control. After the last exposure condition media was measured using Luminex for inflammatory cytokines and growth factors. *p < 0.05, **p < 0.01, and ***p < 0.001 vs. control, N = 3–12 treated wells per group.

In BEAS-2B cells, exposure to Cool Cucumber aerosol resulted in a significant increase in IFN-γ, MCP-1, PDGF-bb, and FGF-basic compared to its unexposed counterparts. Exposure to Classic Menthol resulted in a significant increase in IL-1β, IFN-γ, IL-17, MCP-1, PDGF-bb, and FGF-basic levels compared to the unexposed control group. While Just Mango (Strawberry Coconut) did not cause significant changes in inflammatory mediators, Caffé Latte significantly elevated IFN-γ, MCP-1, and FGF-basic levels compared to the unexposed group. **(**Fig. 5A**)**.

In 16-HBE cells, exposure to Cool Cucumber resulted in a significant increase in IL-8, IL-15, and eotaxin response. Classic Menthol resulted in a significant increase in the production of inflammatory mediators IL-8, IL-15, eotaxin and G-CSF. Exposure to Just Mango (Strawberry Coconut) flavor significantly increased IL-1β, IL-8, IL-15, eotaxin, PDGF-bb, G-CSF, and GM-CSF levels. Caffé Latte exposure significantly increased IL-8, IL-15, PDGF-bb, and GM-CSF responses compared to the unexposed counterparts **(**Fig. 5B**)**. Exposure to pod flavors slightly increased TNF-α levels, Just Mango (Strawberry Coconut) flavor being the highest inducer of the tested flavors **(**Fig. 5B**)**.

### Epithelial barrier function altered by exposure to JUUL pod aerosols

**Figure 6:**
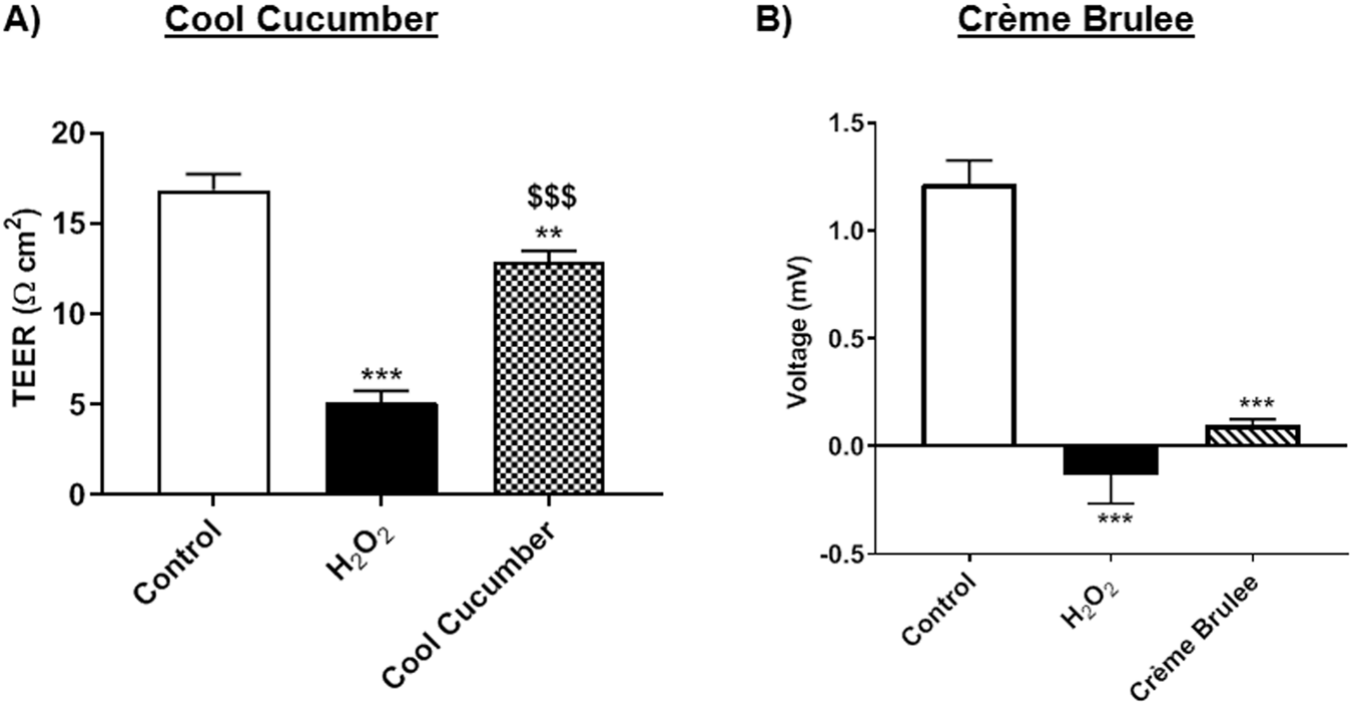
JUUL pod flavors affects barrier function in epithelial cells. (**A**) 20,000 16-HBE cells grown in 24-well transwell plates exposed to three-sessions (each 66 puffs) of JUUL pod flavor cool cucumber with equal intervals of 12 hours between sessions. After the final exposure, the resistance was measured by EVOM2 device. ^$$$^P < 0.001 vs. H_2_O_2_ and, **p < 0.01**p < 0.01, and ***p < 0.001 vs. Control. N = 3–12 wells per group. (**B**) 20,000 16-HBE cells were cultured in 24-well transwell plates. Designated wells were treated H_2_O_2_ (600 μM) for comparison control. JUUL pod, Crème Brulee, was aerosolized using Scireq Inexpose system. Cells were exposed to 66 puffs of Crème Brulee for 30 minutes each day for three consecutive days. Twenty-four hours post-exposure the voltage was measured by EVOM2 device. ***p < 0.001 vs. Control. N = 3–12/group.

To assess the epithelial barrier dysfunction by exposure to various pod flavors, the resistance and the voltage in 16-HBE were measured subsequent to aerosol exposure. Cells exposed to Cool Cucumber resulted in a significant reduction in membrane resistance. Greater reduction in epithelial resistance was observed in cells treated with H_2_O_2_ **(**Fig. 6A**)**. Exposure of 16-HBE to Crème Brulee resulted in a significant reduction in membrane voltage, similar to results seen with the exposure to H_2_O_2_ **(**Fig. 6B**)**. Comparable reductions in TEER measurement was also observed when the 16-HBE cells were treated with 1.0% Crème Brulee liquid. These data in TEER and voltage measurements demonstrate that exposure to JUUL pod flavoring chemicals causes epithelial barrier dysfunction.

### DNA damage caused by JUUL pods and other pod flavors

**Figure 7:**
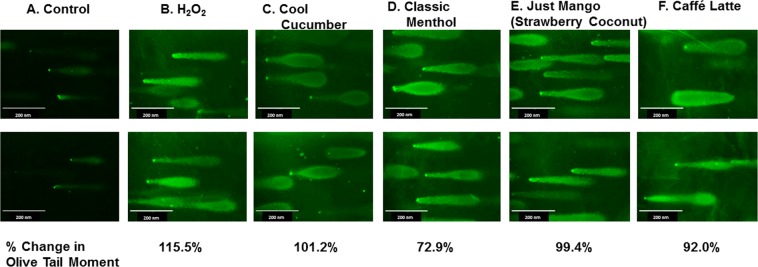
DNA damage by pod flavor liquids. Approximately 300,000 U937 cells cultured in a 12-well plate were treated with 0.5% of selected JUUL pod and other pod flavors, Cool Cucumber, Classic Menthol, Just Mango (Strawberry Coconut), and Caffé Latte. H_2_O_2_ treatment as a comparison group. After 24 hours, cells were prepared for Comet Assay. Fluorescent images were captured to visualize DNA damage. Calculated percent changes in olive moments were presented compared to the untreated control. N = 6 images per sample.

Based on the images from the Comet assay, treatment with H_2_O_2_ has increased the tail lengths compared to the untreated control. On average, the percent change in the olive moment was increased from 72.9% to 101.2% with Cool Cucumber, Classic Menthol, Just Mango (Strawberry Coconut), and Caffé Latte pod e-liquid treatments. As expected H_2_O_2_ caused the greatest change in olive tail moment with a 115.5% increase. These data suggest that exposure to these aerosols can cause significant DNA damage **(**Fig. 7**)**.

## Discussion

The high prevalence of vaping among adolescents has led the Food and Drug Administration (FDA) to issue warning letters and to penalize the stores selling these products to minors^5,12^. However, there is still minimal regulation enforced on e-liquids and vape products. With the escalated use in e-cigs, such as pod mod devices and CBD/THC based formulations for vaping, it is pivotal to assess the toxicological and health effects of these products.

The focus of our current study was on the oxidative stress, inflammatory response, epithelial barrier function, and DNA damage by JUUL pods and other pod flavors. In this study, we tested seven JUUL pods and two other pod flavors in acellular and cellular assays to understand the effects of acute exposure. A recent study demonstrated that JUUL pods and “JUUL-a-likes pods” contain between 1.7% to 7.0% nicotine^13^. Another study has shown total concentrations of flavoring chemicals to be between 0.2 to 15.6 mg/ml and 0.1 to 9.1 mg/ml in JUUL pod liquid and aerosol phases respectively^11^. They identified high levels of menthol, ethyl maltol, and vanillin in JUUL pods^11^. These flavoring chemicals, which have numerous alcohols and aldehyde compounds, react and result in the perpetual formation of byproducts including acetals, such as vanillin PG acetals and vanillin VG acetals^14^. These products induce irritation of the lung and inflammatory responses. Moreover, our previous studies have shown that these flavoring chemicals can induce significant ROS release, oxidative stress, inflammation, and barrier dysfunction^3,15^. Although our findings have seen cytotoxicity in direct treatment in e-liquid of Classic Menthol, JUUL labs have conducted preliminary studies on aerosol exposures with 5% Classic Menthol had no observable cytotoxicity based on neutral red uptake, this difference could be due to the treatment method and cell lines used for assays [TSRC, Tob. Sci. Res. Conf., 2018, 72, abstr. 053].

Corroborating our data comparing ROS in JUUL pods and 3R4F cigarettes, other studies conducted on JUUL pod and other e-cigarette aerosolized vapors showed the production of harmful oxidants, such as free radicals and reactive carbonyls, although the concentration of these oxidants was lower than that of conventional cigarettes^16,17^. In another study, it was determined that nicotine concentration did not have an effect on ROS generation from vapors of e-cigarettes and that as the ratio of VG to PG increased there was a decrease in ROS generation^18^. Although the seven different JUUL pod flavors were able to generate ROS, flavors like Cool Mint, Crème Brulee, Fruit Medley, and Virginia Tobacco were not capable of reducing acellular total glutathione levels (Supplementary Fig. S2). However, studies are required to determine the redox status of the cells by determining intracellular GSH/GSSG levels.

Our data showed increased mitochondrial superoxide production in all tested flavors, including JUUL pods and other pods. The pivotal role of mitochondrial superoxide in driving pro-inflammatory cytokine production in chronic diseases has been well established^19^. This suggests that chronic exposure to JUUL pods and similar pod vapors may cause chronic inflammation via mitochondrial dysfunction in the lung. Consistent with our findings, in non-asthmatic human airway smooth muscle cells exposed to cigarette smoke extract led to increased mitochondrial ROS^20^.

We observed differential inflammatory responses by epithelial cells and monocytes depending on the flavor. Overall, there was an increased level of IL-8, a major neutrophil chemotactic factor, in lung cells exposed aerosols, JUUL pod aerosols (Cool Cucumber and Classic Menthol) and other pod aerosols (Just Mango-Strawberry Coconut and Caffé Latte). Moreover, we also observed that nicotine treatment did neither induce significant IL-8 production nor mitochondrial ROS production in epithelial cells (data not shown). JUUL pod also contains salts along with nicotine and PG/VG. It may be possible that PG/VG and salts with or without nicotine may cause oxidative stress and inflammatory responses which require investigation.

Based on these observations, flavoring chemicals present in these JUUL pods and other pod products may be responsible for the oxidative stress and inflammatory responses elicited in lung cells. Analysis of constituents in JUUL pods by GC-MS showed a wide range of flavoring chemicals, i.e. vanillin, menthol, and cyclohexanols, which are known to cause adverse health effects. Our previous work has shown that the flavors and flavoring chemicals in e-liquids can induce significant oxidative stress and inflammatory responses in lung cells^3^.

IL-8 stimulates neutrophil migration across endothelium and can activate its functions, including respiratory burst and free radical production. Excessive acute inflammation can result in lung injury, dysregulated repair, and impair gas exchange^21^. Significant increases in IL-8 have been observed in sputum and bronchoalveolar lavage fluid (BALF) of patients with pulmonary diseases such as asthma, idiopathic pulmonary fibrosis, and chronic obstructive pulmonary disease^22^. Based on recent case reports, PG/VG and flavorings used in JUUL pods and other pods especially the formulations with CBD/THC MCT oil with terpenes and vitamin E (alpha-tocopherol) acetate peroxyl radical formation may cause acute lung injury via foamy/lipoid (lipid-laden) macrophages leading to lipoid/hypersensitivity pneumonia and/or chemical-induced injuries, which requires investigation^23^. Further, endogenous phospholipids or surfactants breakdown by vaporized e-liquid/juices containing THC/CBD MCT oil along with terpenes and PG/VG can cause a similar response. Prostaglandin E_2_ (PGE_2_), an arachidonic acid-derived mediator through cyclooxygenase, is associated with pro-inflammatory cytokine response in general. PGE_2_ is involved in the homeostasis of immune-inflammatory response and lung remodeling process^24^. The increased PGE_2_ production observed in monocytes and epithelial cells with certain flavors may suggest that this homeostasis has been affected by the flavor constituents.

Consistent with the aforementioned IL-8 levels, we also observed elevated concentrations of other major pro-inflammatory mediators, such as IL-1β and IFN-γ by epithelial cells. Elevated IL-1β levels are known to cause pulmonary inflammation as observed in emphysema and airway remodeling in mice^25^. Correlating with this data, IFN-γ is a potent stimulator of CC and CXC chemokines. Cigarette smoke has been shown to induce pulmonary inflammation and emphysema via IFN-γ dependent pathways^26^.

GM-CSF levels were increased with Just Mango (Strawberry Coconut) and Caffé Latte pod flavor exposures. These differential responses may be attributed to the interplay between PGE_2_ and GM-CSF as there have been studies demonstrating regulation of fibrogenesis by GM-CSF mediated PGE_2_ levels^27^. Studies on GM-CSF have shown that BEAS-2B cells exposed to conditioned media that had e-cigarettes bubbled through it had a significant increase in GM-CSF levels^28^. We observed increased G-CSF levels in epithelial cells after Classic Menthol and Just Mango (Strwarberry-coconut) pod flavors exposure. In the BALF of COPD patients increased levels of G-CSF have been found^29^. Furthermore, deletion of G-CSF has been linked to reduced airway inflammation and tissue destruction^29^. These suggest that G-CSF may play a vital role in the pathogenesis of pulmonary diseases. Growth factors such as PDGF have been shown to displays chemoattractant properties at low concentrations for neutrophils, macrophages, and fibroblasts and can also display fibroblast stimulating properties. FGF has been shown to display proliferation properties but also can regulate the synthesis and deposition of extracellular components^30^. The observed upregulation of growth factors such as PDGF has been implicated as players in cellular proliferation and angiogenesis^31^. A recent study, bolstering our findings, showed that repetitive inhalation to e-cigarettes with nicotine could induce pro-fibrotic and pro-inflammatory mediators, such as angiopoietin-1/2, EGF, LIF (IL-6 member), and LIX (IL-8 homolog), in C57BL/6 and CD-1 mice^32^.

All tested pod flavors induced IL-15, a mediator for T-cell and NK cell-mediated inflammation, indicating the risk of chronic lung inflammation in the lungs with exposure to these flavor constituents^33,34^. Additionally, in accordance with the above findings, observed significant increases in eotaxin and MCP-1 also suggest eosinophilic and monocytic inflammatory processes in the lung due to flavor inhalation exposure.

Our data on epithelial barrier function show that in 16-HBE cells, exposure to JUUL pod Cool Cucumber resulted in a significant decrease in normalized resistance and exposure to JUUL pod Crème Brulee resulted in a significant decrease in membrane voltage. Other studies have shown that repeated exposure to cigarette smoke increased the epithelial monolayer permeability, and a reduction in adhesion proteins, E-cadherin and β-cadherin^35^. Other studies on the effects of nicotine and e-cigarette chemical flavorings on epithelial barrier function have found that both *in vitro* exposure to nicotine and flavoring chemicals such as diacetyl and coumarin resulted in a significant reduction in normalized resistance to the epithelial barrier function of 16-HBE^15^. Consistent with other studies, our results show that JUUL pod vapors resulted in decreased normalized resistance and voltage in the epithelial membrane. Vapor induced barrier dysfunction characteristics are in corroboration with elevated pro-inflammatory mediators, such as IL-1β and IFN-γ, which are known to play an intimate role in airway junctional disintegration in epithelial cells^36^. Epithelial barrier dysfunction can lead to acute lung injurious response by epithelial leakage. Other studies have shown cystic fibrosis transmembrane conductance regulator (CFTR) and calcium-activated chloride channels (CaCCs) dysfunction induced by acute e-cig vapor^37^. These adverse effects can induce thickening of the mucus and impaired mucus clearance as observed in COPD patients^37^.

We observed DNA damage caused by JUUL pod flavors with increasing DNA fragmentation with aerosol exposures. These data are consistent with other studies where e-cigarette exposure caused 8-hydroxy-2′-guanosine, O^6^-methyldeoxyguanosine, and γ-hydroxy-1,N^2^-propano-deoxyguanosine adduct formation in the lung tissue^38,39^. DNA damage can lead to increased cellular senescence and the release of inflammatory mediators by cellular secretory phenotype.

Furthermore, the risk of cardiovascular diseases (CVD) should not be ignored as the constituents of pod based devices are similar to that of other e-liquids, with high nicotine concentrations, aldehydes, oxidants, particulates, and flavor chemicals. Inhalation of these flavors potentially similar CVD risks, including atherosclerosis, hypertension, thrombogenesis, and myocardial infarction^40^.

In conclusion, our data show that JUUL and other pod flavors produce cellular ROS, triggering an inflammatory response, epithelial barrier dysfunction, and DNA damage in lung cells. Further studies are required to test pod based devices in inducing lung cellular stress and toxicological responses *in vivo* and understand the mechanisms involved by flavoring chemicals. This may provide information on flavoring chemical-induced lung injury, leading to the regulation of inhalable flavoring chemicals in pods and e-liquids/e-juices.

## Supplementary information

Supplementary Information

## Data Availability

We declare that we have provided all the data, but the primary data will be available upon request.
