# Supplementary material for: E-cigarette flavored pods induce inflammation, epithelial barrier dysfunction, and DNA damage in lung epithelial cells and monocytes
Source: Sci Rep. 2019 Dec 13;9:19035. doi: 10.1038/s41598-019-51643-6 (PMC6910911; doi:10.1038/s41598-019-51643-6)
Supplement: Supplementary file 1 — Supplementary Information [file 41598_2019_51643_MOESM1_ESM.pdf]

Supplementary Information

E-cigarette flavored pods induce inflammation, epithelial barrier dysfunction, and DNA damage in lung epithelial cells and monocytes

*Thivanka Muthumalage, *Thomas Lamb, Michelle R. Friedman, and Irfan Rahman

*These authors contributed equally to this work

Supplementary Table S1: Chemical composition of JUUL pod flavors, Fruit Medley, Classic Menthol, Cool Mint, Crème Brulee, Cool Cucumber, Mango, and Virginia Tobacco, presented in retention time and percent probability in parenthesis

Chemical Compound	CAS#	Fruit Medley	Classic Menthol	Cool Mint	Crème brulee	Cool Cucumber	Mango	Virginia Tobacco
heptaethylene glycol	5617-32-3					5.318 (14.7)	5.324 (15.1)	
butanedioic acid, 2,3-dimethoxy-, diethyl ester	53431-90-6	5.336 (19.1)		5.330 (11.4)	5.324 (16.2)			5.324 (10.5)
butanoic acid, 4-(1,1-dimethylethoxy)-3-hydroxy-, methyl ester, (R)	133-32-4	5.582 (26.9)						
sulfide, sec-butyl isopropyl-	22438-36-4				5.662 (15.9)			
sorbitol	50-70-4						5.736 (10.2)	
3-ethoxy-1,2-propanediol	1874-62-0				5.851 (22.4)			
benzyl alcohol	100-51-6			5.925 (44.7)				
2-propanol, 1,1'-oxybis-	110-98-5	5.994 (49.4)				5.873 (49.7)		
cis-beta-terpineol	7299-41-4			6.125 (22.8)				
cyclohexanol, 1-methyl-4-(1-methylethyl)-(MENTHOL DERIVATIVE)	21129-27-1	6.823 (15.1)	6.812 (17.4)			6.812 (14.6)		
benzoic acid	65-85-0	7.298 (45.1)	6.932 (31.1)	7.087 (41.9)	7.012 (38.7)	7.075 (45.9)	6.921 (38.1)	6.966 (45.7)
glycerin	247035-47-8	7.911 (85.5)	6.440 (85.4)	6.686 (82.7)	6.697 (73.8)	6.440 (85.5)	6.509 (75.1)	6.589 (73.0)
			7.041 84.3)			7.458 (83.7)	6.995 (84.3)	7.138 (83.2)
menthol *	89-78-1	6.823 (12.2)	6.812 (12.6)	6.823 (11.3)	NO	6.812 (10.3)	NO	NO
desulphosinigrin	5115-81-1						7.956 (20.0)	9.541 (8.07)

Chemical Compound	CAS#	Fruit Medley	Classic Menthol	Cool Mint	Crème brulee	Cool Cucumber	Mango	Virginia Tobacco
1,2,3-propanetriol, diacetate	102-62-5			8.031 (67.5)				
pyridine, 3-(1-methyl-2-pyrrolidinyl)-, (S) -(NICOTINE)	54-11-5	8.300 (80.3)	8.219 (81.0)		8.237 (81.4)	8.254 (80.8)	8.220 (79.6)	8.236 (80.5)
vanillin	121-33-5				8.569 (67.3)			
2(3H)-furanone, 5-hexyldihydro-	706-14-9	9.026 (39.7)						
benzaldehyde, 3,4-dimethoxy-	120-14-9			9.547 (24.4)	9.290 (75.3)			9.066 (79.4)
pyridine, 3-(2-piperidinyl)-, (S)-	494-52-0	10.285 (32.2)						
DL-xylitol, 1-benzoate	13078-04-1	10.629 (14.3)						
benzoic acid, hexadecyl ester	22485-54-7				11.372 (8.19)			
benzoic acid, pentadecyl ester	56954-74-6				11.401 (8.06)			11.372 (11.5)
								11.401 (14.2)
hexadecanoic acid, methyl ester	112-39-0				11.452 (53.9)	11.447 (69.8)	11.447 (74.7)	11.452 (72.6)
						12.276 (69.8)		
terephthalic acid, 2-nitro-5-sulfonyl-	30211-77-9				11.533 (9.18)			
9-octadecenoic acid (Z)-, methyl ester	112-62-9	12.196 (7.90)						
10-octadecenoic acid, methyl ester	13481-95-3	12.225 (12.7)			12.191 (8.27)			12.196 (6.57)
octadecanoic acid, methyl ester	112-61-8	12.282 (67.4)			12.276 (46.7)		12.276 (58.0)	12.282 (52.9)

Chemical Compound	CAS#	Fruit Medley	Classic Menthol	Cool Mint	Crème brulee	Cool Cucumber	Mango	Virginia Tobacco
phenol, 2,4-bis(1,1-dimethylethyl)-	96-76-4							9.255 (22.6)
benzene, 4-(dimethoxymethyl)-1,2-dimethoxy-	59276-33-4							9.598 (79.4)
2(3H)-furanone, 5-heptyldihydro-	104-67-6						9.690 (78.5)	
ethyl citrate	77-93-0						10.148 (89.8)	10.153 (93.7)
octadecane, 3-ethyl-5-(2-ethylbutyl)-	55282-12-7							10.428 (6.68)
heptacosane	593-49-7							10.651 (6.76)
tetradecane, 2,6,10-trimethyl	14905-56-7							10.743 (11.5)
diphenyl sulfone	127-63-9							11.618 (45.8)
heptacosane	593-49-7							11.647 (8.96)
squalene	111-02-4				15.675 (16.9)			

Supplementary Figure 1

A: Internal structure of a JUUL pod

A JUUL pod consists of a stainless steel vapor path, a silica wick, and a nichrome coil heater enclosed in heat resistant plastic and pod covers. (Authors took JUUL pods apart themselves and took pictures)

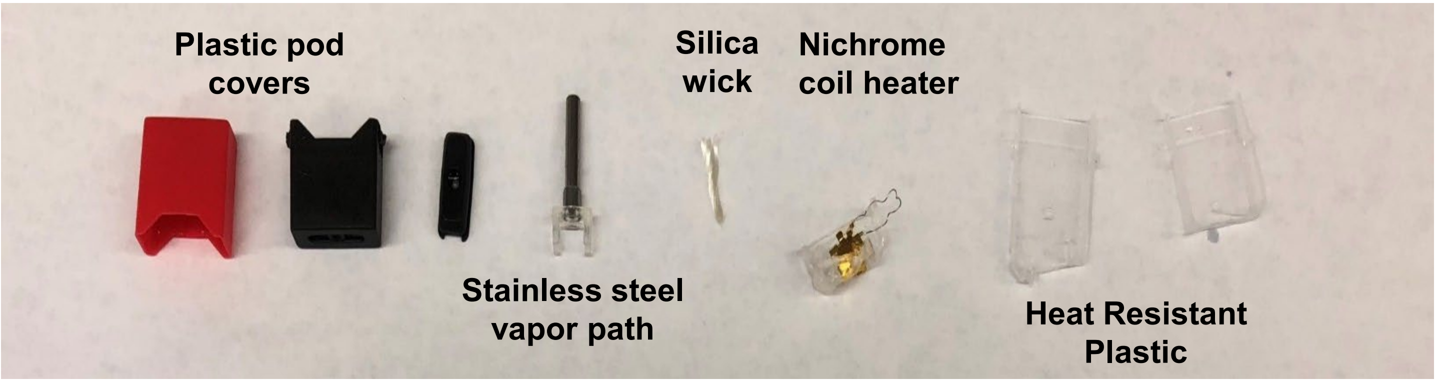

B. In-vitro exposure setup

JUUL pod device connected to Scireq Inexpose pump delivering three puffs/min, with 55 mL puff volume over 22 minutes (66 total puffs), to the Enzyscreen chamber containing cell culture plates. Cells were allowed an additional 8 minutes in the chamber with a cumulative 30-minutes during a single aerosol exposure session.
(The authors have drawn this schematic by themselves)

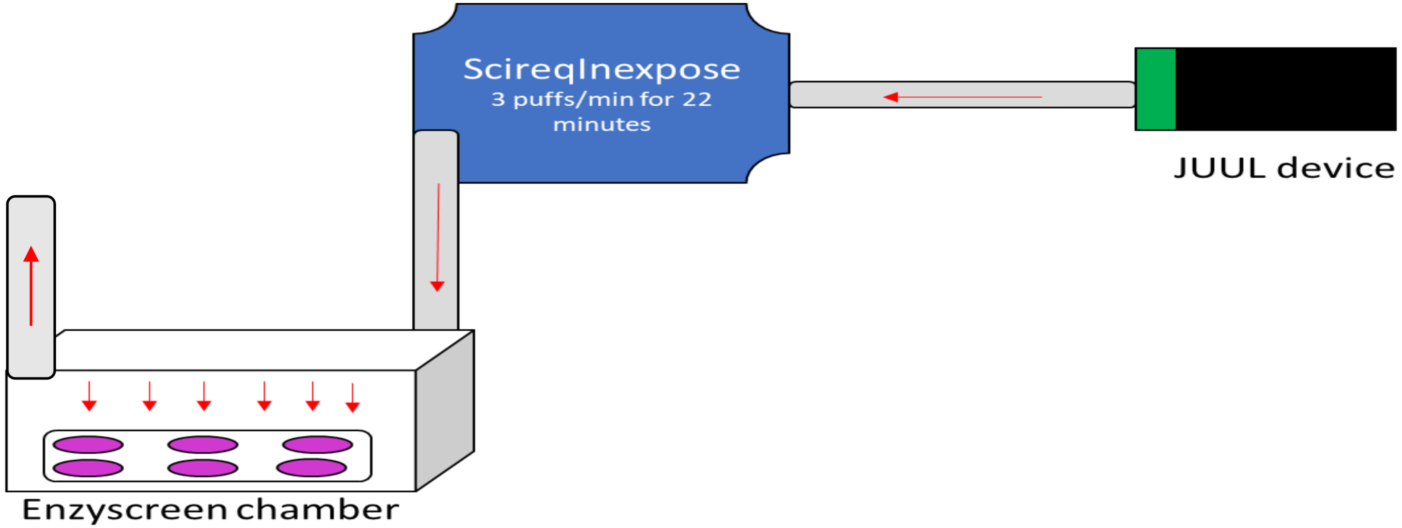

Supplementary Figure 2: Acellular glutathione reduction by JUUL aerosols

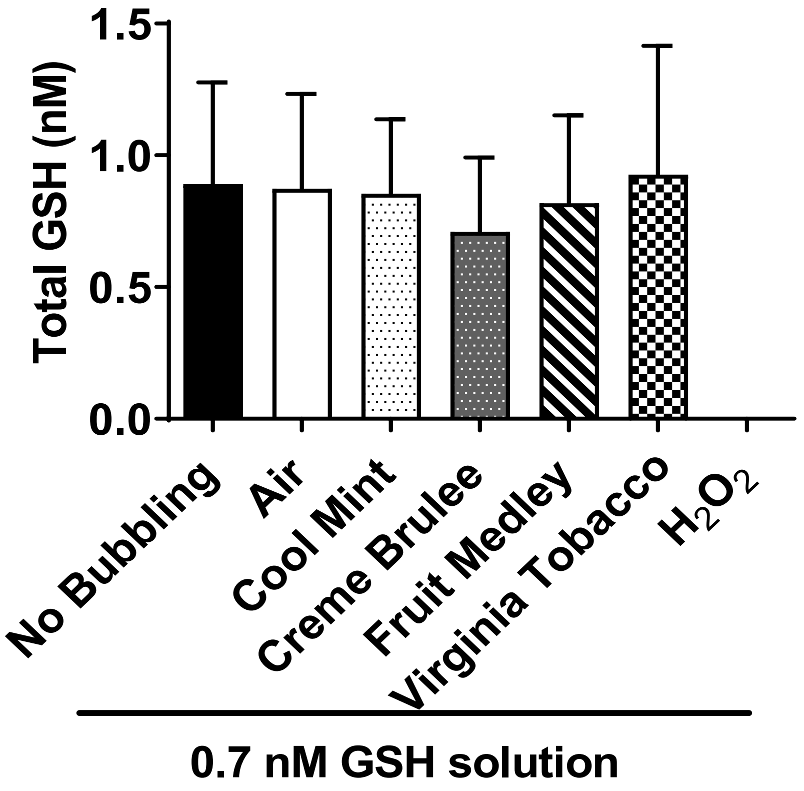

JUUL pod does not affect acellular total glutathione levels

Fifteen puffs of Cool Mint, Crème Brulee, Fruit Medley, and Virginia Tobacco pod flavors were bubbled through 0.7 nM glutathione solution using Scireq Inexpose system with a three-second puff duration. For control comparison control groups, H_2O_2 (30%) was used as the positive control while DCF-dye alone air-bubbled as negative controls.
